# Polymer-Driven and Hybrid Actuation Fabrics: Integrating Responsive Polymeric Materials and Hierarchical Textile Architectures

**DOI:** 10.3390/polym18151881

**Published:** 2026-07-31

**Authors:** Wanyu He, Rujun Yu, Bin Fei

**Affiliations:** School of Fashion and Textiles, The Hong Kong Polytechnic University, Hong Kong, China; wanyu.he@connect.polyu.hk (W.H.); rujun.yu@connect.polyu.hk (R.Y.)

**Keywords:** actuation, functional fabrics, smart textiles

## Abstract

Textile fabrics have served as a second skin for millennia, yet their potential as active engineering systems is only beginning to be realized. Historically, most smart textiles have treated fabrics as passive substrates for sensors, conductors, or rigid motors. A paradigm shift is underway toward intrinsic actuation fabrics, where active polymers—including liquid crystal elastomers (LCEs), twisted and coiled polymer actuators (TCPAs), and shape memory polymers (SMPs), as well as polymeric yarn/fabric matrices integrating shape memory alloys (SMAs), serve as functional engines to generate motion, force, or shape change. Despite rapid progress in functional materials development, a critical gap persists between fiber-level actuation mechanics and fabric-level system implementation. This review addresses that transition by establishing a four-tier hierarchical framework (Fiber, Yarn, Fabric, and System) to clarify how responsive building blocks are structurally integrated. We systematically analyze how traditional textile architectures—including woven, knitted, braided, and non-woven structures—mechanically amplify, redirect, or constrain the intrinsic stroke and force of active polymers and SMA-polymeric hybrids. By bridging recent advances in polymer materials science with textile structural mechanics, this review provides structural design strategies and highlights grand challenges in wearability, durability, and system integration for next-generation polymeric soft actuation fabrics.

## 1. Introduction

For centuries, fabrics have been utilized primarily for protection, comfort, and esthetics. In recent decades, smart textiles have introduced sensing, electronic, and interactive functions; however, many early systems continued to rely on a substrate-based design, with textiles serving mainly as support for externally added components [1]. In prehistoric and early historical periods, textiles functioned as passive materials. Fibrous materials were employed for clothing, ropes, baskets, tents, and shelters, leveraging their flexibility and tensile strength were exploited rather than active capabilities [2,3]. Traditional technical textiles also exploited mechanical performance, yet the textile typically remained a passive load bearing or covering element [3,4]. Similarly, textiles used in transducers have often acted as flexible and breathable bases for electronic components, sensors, and conductors, facilitating e-textiles and wearable devices [5,6,7]. Although this approach expanded textile functionality, it did not fully utilize the intrinsic mechanical programmability of textile structures.

Recent reviews highlight the development of smart, interactive, and electronic textiles, where fibers contribute to sensing, actuation, energy harvesting, and computation [1,3,8,9,10,11,12]. The subsequent advancement is intrinsic textile actuation, which aims not only to simply attach a machine to a garment, but to make the textile itself perform mechanical work. In this paradigm, active textile materials and structures convert thermal, electrical, pneumatic, or environmental stimuli into deformation.

The rationale for this shift is evident. Conventional rigid actuators are frequently heavy, bulky, and unsuitable for close contact with the human body. In contrast, textiles provide compliance, breathability, softness, and structural adaptability. Fabrics composed of numerous interacting yarns capable of sliding, rotating, and redistributing stress, tolerate complex deformation and resist damage more effectively than continuous films or rigid sheets [13,14,15,16]. Integrating inherent textile properties with active material behavior enables new applications in rehabilitation, soft robotics, adaptive garments, and wearable interfaces. Textiles thus represent promising platforms for wearable robots that move and feel similar to everyday clothing. Their hierarchical structure also offers multiple levels of programmability, from fiber composition and yarn architecture to fabric construction and garment form.

Although recent reviews have established actuation textiles as promising platforms for smart wearables and soft robots because they combine mechanical compliance, programmability, and application breadth, they are still organized mainly around functional materials, stimulus mechanisms, performance improvement, or application scenarios rather than around textile architecture itself [17,18,19]. This emphasis has advanced the understanding of material classes and driving modes, yet it leaves comparatively underdeveloped a structure-led view of how textile formation governs anisotropy, deformation mode, force transmission, body conformity, and system integration, even though recent work explicitly notes that textile-forming technologies strongly shape the morphology, size, mechanical behavior, and actuation performance of wearable textile actuators [18,19]. In particular, textiles are not merely passive carriers for active matter: their intrinsic hierarchy enables preprogrammed architectures, and actuation in many textile systems emerges largely from structural reconfiguration across fiber, yarn, and fabric assemblies [19]. Motivated by this gap, the present review adopts a textile-structure-centered mainline and proposes a four-tier hierarchical system—Fiber, Yarn, Fabric, and System—to unify diversified active materials, textile-forming methods, and actuation modes within a single multiscale framework [19]. This perspective shifts the discussion from a catalog of responsive materials toward the structural logic by which actuation is amplified, constrained, programmed, and ultimately translated into wearable and robotic function.

## 2. Diversified Textile Structures for Actuation

The movement of an actuation fabric depends not only on the material from which it is made, but also on how the textile is constructed. Textile geometry determines the direction of force transmission, the degree of extensibility, and the resulting deformation mode. In most cases, the behavior of an actuating fabric is not governed by the active material alone, but by the hierarchical textile architecture in which that material is embedded [20,21,22,23]. Textile structures can be classified according to dimensionality and interlacing method, ranging from one-dimensional fibers and yarns to two-dimensional fabrics and three-dimensional textile forms. Knowing these structural features is essential for programming actuation direction, force, and amplitude.

**Figure 1:**
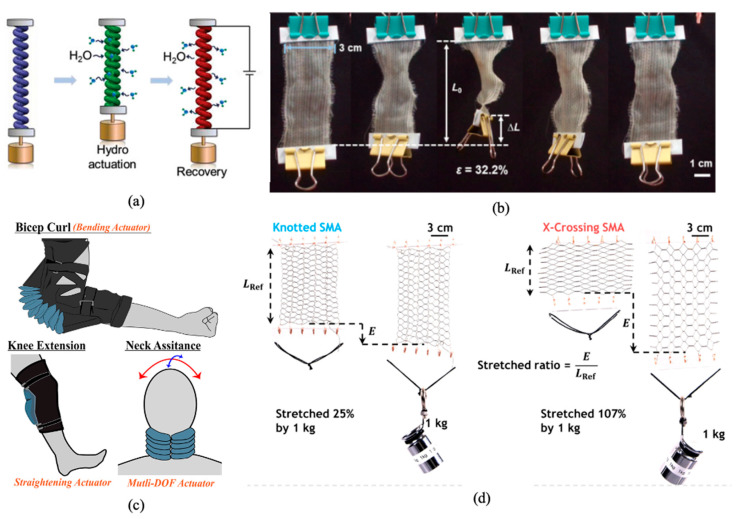
Structural diversity and mechanical behaviors of yarn- and fabric-level actuators across representative textile form factors. (**a**) Twisted and coiled conductive polymer yarn actuator responding to humidity, illustrating how asymmetric swelling and shrinkage in a helical yarn can convert environmental stimulus into torsional contraction and macroscopic motion. Reprinted with permission from ref. [31], copyright 2024, Wiley-VCH GmbH. (**b**) Electro-active liquid crystal elastomer (LCE) actuation fabric showing tensile strain response and large-scale deformation, highlighting how fabric architecture can amplify material-level actuation into textile-scale shape change. Reprinted with permission from ref. [32], copyright 2026, American Chemical Society. (**c**) Structured pneumatic fabric actuators integrated into wearable assistance systems, demonstrating how textile constraints, inflatable elements, and garment form factors can be combined to achieve practical bending and load-bearing functions in wearable settings. Reprinted from ref. [29] under the Creative Commons CC BY license. (**d**) Comparative deformation profiles of knotted and X-crossing shape memory alloy (SMA) fiber meshes subjected to a static 1 kg hanging load, showing how inter-fiber topology and connection pattern influence load redistribution, stability, and overall deformation mode. Reprinted from ref. [33] under the Creative Commons CC BY license.

Fabrics in actuator systems can function either as passive structural elements or as active components. In soft pneumatic actuators, fabrics are commonly used as shells, constraint layers, or strain-limiting substrates that guide inflation and deformation [24,25,26,27,28,29] (Figure 1c). By contrast, in actuation fabrics, textile elements such as active fibers, yarns, or fabric architectures participate directly in force generation and shape change [27,30].

### 2.1. The Foundation: Fiber- and Yarn-Level Structures

**Figure 2:**
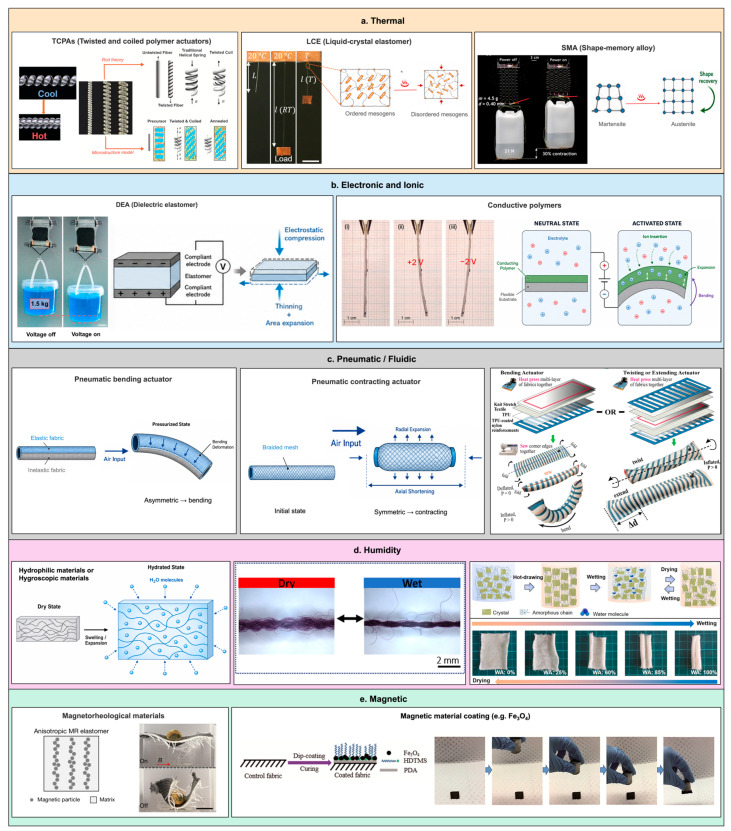
Material classification, actuation mechanisms, and structural motion modes of active fibers and fabrics categorized by driving stimuli. (**a**) Thermal actuation: Twisted and coiled polymer actuators (TCPAs) generating contraction through anisotropic thermal expansion and helical unwinding, which is different from simple helical structure (adapted with permission from ref. [34], copyright 2014, AAAS; and ref. [35] under the Creative Commons CC BY license); liquid-crystal elastomers (LCEs) undergoing reversible axial contraction triggered by order-disorder mesogen phase transitions upon heating (adapted with permission from ref. [77], copyright 2021, AAAS); shape-memory alloys (SMAs) recovering memorized shapes via temperature-induced martensite-to-austenite phase transformation (adapted with permission from ref. [33], copyright 2026, AAAS). (**b**) Electrical and Ionic actuation: Dielectric elastomer actuators (DEAs) operating via electrostatic Maxwell stress-induced compression and area expansion under high voltage (adapted from ref. [78] under the Creative Commons CC BY license); conducting polymer actuators driven by electrochemically induced ion insertion/extraction causing reversible volume swelling and asymmetric bending (adapted from ref. [62] under the Creative Commons CC BY license). (**c**) Pneumatic and Fluidic actuation: Pneumatic and fluidic actuators make use of the different characteristics of various textile fabrics to enable the realization of various deformations, such as bending, shrinking, twisting, etc. Asymmetric fabric-reinforced pneumatic actuators achieving directional bending deformation; braided soft actuators converting radial expansion into axial contraction; layered active textile architectures programmed for complex bending, twisting, or extending under internal pressurization (adapted from ref. [29] under the Creative Commons CC BY license). (**d**) Humidity-induced actuation: Moisture-responsive smart fibers and fabrics driven by water molecule absorption/desorption, where differential swelling or hydrogen-bond breaking triggers reversible yarn contraction and macro-scale structural shape-morphing. The light microscope image shows the woolen yarn at dry and wet states, demonstrating a water-sensitive shape memory effect (adapted with permission from ref. [52], copyright 2021, ACS). Schematics and photographs on the right illustrate the proposed mechanism of secondary polymer structure response to hot-drawing and wetting, alongside the macro-scale shape-morphing of a knitted fabric muscle induced by varying water absorption levels (WA: 0% to 100%) (adapted with permission from ref. [79], copyright 2021, ACS). (**e**) Magnetic actuation: Magnetorheological elastomers and functional magnetic-coated textiles exhibiting rapid, non-contact reconfiguration and localized stiffness tuning guided by external magnetic field distribution; optical image on the left demonstrates a plain-weave magnetorheological (MR) fabric exhibiting stiff–soft duality under magnetic fields (adapted from ref. [80] under the Creative Commons CC BY license). Schematics and photographs on the right illustrate the preparation procedure and real-time magnetic manipulation of a superhydrophobic Fe_3_O_4_-coated cotton fabric (adapted from ref. [81] under the Creative Commons CC BY license).

At the most fundamental level, textiles begin with fibers that are assembled into yarns. Research has shown that twisting polymer fibers, such as nylon fishing line or polyester sewing thread, can convert them into twisted and coiled polymer actuators (TCPAs) [34], as schematically illustrated in Figure 1a. These artificial muscles use helical geometry and stored twist to produce contraction or expansion under stimulus, but they are different from simple helical structures, which are shown in Figure 2a. Since the work of Haines et al. in 2014, TCPAs have attracted broad interest in soft robotics and smart textiles because they are lightweight, low-cost, scalable, flexible, and capable of producing large strokes and forces [34,35]. Beyond the early use of nylon and polyester, researchers have explored carbon nanotube yarns, shape memory materials, natural fibers, liquid crystal elastomer (LCE) fibers, and composite systems for fiber- and yarn-level actuation [35,36,37,38,39,40,41].

Beyond TCPAs, straight fiber or actuation yarns remain common basic structures [42], and bilayer or Janus fiber actuators have also been developed [43]. Actuation yarns can be mainly classified by their compositions. One kind of actuation yarn is fully made of actuation fibers, while others blend with passive fibers to save cost or change actuation motions. Overall, yarn-level actuation provides the active building blocks for more complex actuator systems based on textile materials.

### 2.2. Woven Structures: Stability and Force

At the two-dimensional level, fibers and yarns are assembled into fabrics, which brings actuators based on textile materials closer to practical application. Fabric actuators may be woven, knitted, non-woven, braided, or constructed using other specialized architectures. In these systems, the fabric may either form the actuator body, as in pneumatic chambers, or host active yarns such as shape memory alloy (SMA), LCE, or coiled polymer fibers that generate motion. Woven fabrics are one of the major structural categories in actuation fabrics. Weaving interlaces warp and weft yarns at approximately right angles, producing stable structures such as plain, twill, and satin weaves [4].

The plain weave is the most basic woven structure, in which warp and weft yarns alternately pass over and under each other [4]. Its high number of interlacing points gives the fabric strong dimensional stability and relatively low extensibility. In actuation fabrics, this stability is useful when high force output or directional constraint is more important than large shape change. The warp-weft architecture provides anisotropy and scalability, while embedded active yarns or internal bladders generate bending, contraction, or twisting under external stimuli.

As noted above, woven fabrics can act either as passive substrates or as active elements. In passive systems, woven fabrics mainly serve as shells or constraint layers for internal bladders in soft pneumatic actuators [44,45,46,47]. For example, Zhang and colleagues developed a woven fabric that enabled powerful and high-range pneumatic actuation. In their design, TPU-coated plain woven fabric provided air tightness and heat-sealing capability. The dense woven structure prevented uncontrolled balloon-like expansion under high pressure, allowing the actuator to achieve higher load-bearing capacity and stiffness than many conventional elastic soft actuators [48]. More recently, textile materials have increasingly been used as active actuation elements. Yang et al. created multifunctional soft robots by weaving liquid crystal elastomer actuation fibers. The woven architecture provided flexibility and multi-degree-of-freedom motion, showing how traditional textile construction can be used to organize active fibers into functional robotic systems [49].

Although plain weave is the most widely used woven structure, other patterns such as twill and satin have been less explored in actuation fabrics. More complex structures, including jacquard and multilayer weaves, also remain underused. Recent work by Zhang and colleagues investigated how weaving patterns affect the performance of LCE fibers (Figure 1b). A single LCE fiber mainly contracts along one direction, whereas weaving it into a two-dimensional fabric enables deformation at the fabric scale. By comparing different woven patterns, the authors found that the number and distribution of interlacing points strongly influence actuation performance. Satin weave, with more widely spaced crossing points, allowed LCE fibers to contract with less constraint, achieving area shrinkage of over 42% and enhanced pulling force [32].

By selecting weave patterns such as twill or satin, researchers can create anisotropic actuation, where a fabric curls or contracts preferentially in one direction while remaining more stable in another. In this way, weave architecture becomes a tool for programming deformation, rather than merely a method for holding active yarns together.

### 2.3. Knitted Structures: Compliance and Strain Amplification

In contrast to weaving, knitting makes one or more yarns into connected loops. This interloop architecture produces structures that are inherently stretchable and conformable, even when the yarn material itself is relatively stiff. Basic knitted structures include jersey and rib, while stitch types such as knit, tuck, and float can be combined to generate diverse patterns. Other common structures include interlock, Milano, cardigan, and intarsia. With computerized flat-bed and circular knitting machines, more advanced forms such as jacquard fabrics and three-dimensional knitted structures can also be produced.

The structural elasticity of knitting is a key design feature for soft robotics. When active materials such as SMA wires are knitted, the resulting fabric does not simply contract linearly; instead, the loop network collapses, rotates, and redistributes strain. Compared with dense woven structures, which often restrict actuator strain, knitted structures can amplify deformation through loop rearrangement.

Two-dimensional knitted fabrics are often anisotropic because their loop geometry makes them stiffer in one direction and more extensible in another [44]. Like 3D printing, knitting can shape materials into designed structures and patterns. However, textile forming offers additional advantages, including open internal spaces, reduced need for support materials, and fewer concerns about interfacial adhesives or chemical compatibility between components. During knitting, yarns are mechanically held within the structure, often without requiring bonding agents. The challenge is that programming a knitting machine is complex: the action of each needle must be controlled precisely, and successful fabrication usually requires expertise in pattern design and machine operation [1]. This complexity has limited the use of knitting in transducer and robotics research. Nevertheless, researchers are increasingly incorporating knitting into actuator fabrication. As with woven fabrics, knitted fabrics can serve either as passive substrates or as active actuation components. In passive systems, du Pasquier et al. presented Haptiknit, a haptic sleeve combining machine knitting and pneumatic actuation. The device uses a two-layer knitted fabric, with a stiff outer layer to ground actuation force and a soft inner layer to transfer load to the skin. Although the knitted structure functions mainly as a passive base, it seamlessly integrates different materials without adhesives and combines comfort, tactile feedback, and controllable inflation [26]. Its loop structure also allows the sleeve to conform to the complex curvature of the human body without restricting movement, which is difficult to achieve with stable woven fabrics.

Knitting techniques are also gaining attention for producing active actuation fabrics instead of passive substrates for actuators because of their stretchability and structural complexity. As LCE fibers have emerged as high-performance active materials, their integration into knitted architectures has become an important research direction. Wan et al. demonstrated a circularly knitted LCE tubular textile that converts the one-dimensional response of LCE fibers into biaxial fabric contraction and complex three-dimensional motion [50]. Unlike ordinary LCE tubes, which typically expand radially while contracting axially, the knitted LCE tube can shrink in both the radial and axial directions when heated. This unusual bidirectional shrinkage is valuable for soft robotic systems. Yu et al. further showed that double-network construction with meshed fabrics can strengthen LCE actuation, reinforcing the role of textile architecture in amplifying material response [51]. Likewise, Iqbal et al. demonstrated knit architecture for water-actuating woolen knit-wear and personalized thermal management, highlighting how knitted form can couple actuation with comfort-oriented garment functions [52]. Overall, knitting uses loop architecture to transform fiber-level actuation into two- or three-dimensional textile deformation, enabling amplified strain and programmable behaviors such as bending, twisting, and radial contraction.

Compared with simple cut-and-sew actuation fabrics, advanced knitting is increasingly attractive for wearable rehabilitation because it offers a more direct route from textile structure to device function. High-impact work in Science Advances showed early on that knitting can amplify actuator strain through looped architectures, yielding a 53-fold strain increase relative to the underlying yarn response while preserving mechanical stability, which established knitting as more than a passive substrate for wearable actuation [53]. Later studies in Advanced Functional Materials and Nature Communications reinforced this view by showing that knit mechanics arise from stitch topology, geometric rearrangement, and yarn stretching, so elastic and actuation behavior can be programmed at the stitch-by-stitch level rather than being dictated only by constituent material properties [22,24]. This structural programmability is especially relevant to rehabilitation wearables, where safe human–machine interaction, low apparent stiffness, and conformity to complex body geometry are central design requirements [43].

Within this broader framework, 3D seamless whole-garment knitting provides a particularly strong manufacturing route for rehabilitation devices because it enables actuator-integrated textiles to be produced in a one-piece, low-postprocessing form rather than by attaching discrete actuators to finished garments [24,54]. This matters mechanically as well as practically: seam-free fabrication reduces assembly complexity, lowers bulk, and avoids seam-related discontinuities that can become stress concentrators or failure points under repeated deformation [54]. In wearable rehabilitation, where devices must remain comfortable under prolonged skin contact and repeated joint motion, this shift from actuator-on-textile assembly to textile-native actuation architectures is a meaningful advantage [27,55]. A second advantage of whole-garment knitting is its capacity for anatomically conformal 3D shaping. Self-fitting SMA knit garments have shown that knitted actuator networks can dynamically conform to the unique topography of the wearer’s body, including concave and convex regions that passive fabrics and conventional closure-based fits struggle to accommodate [56]. This is not merely a matter of comfort; many wearable robotic functions depend on simultaneous body proximity and controlled local stiffness, so improved topographical conformation directly affects force transfer, sensing fidelity, and assistance efficiency [56]. A third advantage is the ability of knitted architectures to support more spatially uniform pressure and load transfer. Knitted rehabilitation devices do not simply stretch; their loop networks rotate, collapse, and redistribute deformation, which allows stiffness and extensibility to be tuned locally across the garment [22,24]. This is important for rehabilitation sleeves, gloves, and compression-like assistive systems, because effective assistance usually depends on distributed loading rather than concentrated point forces [26,57]. Haptiknit manufactured by Pasquier demonstrated that distributing high- and low-stiffness knit regions within one sleeve can ground actuator forces while preserving wearability, enabling each actuator to transmit 40 N at 14.5 Hz in a portable textile form factor [26]. More generally, the same principle suggests why seamless knitted rehabilitation garments can produce more homogeneous pressure fields than stitched, strap-based, or discretely mounted systems: local elasticity, actuator zones, and sensing paths can be embedded continuously within the same fabric architecture [55].

Weft knits have dominated most recent soft robotic demonstrations because they provide high extensibility, large geometric rearrangement, and fine local tunability, which are ideal for bending, contraction, and distributed pneumatic actuation [55]. Their deformation mechanics are strongly governed by structural compliance, not just yarn stiffness, which makes them well suited to large-amplitude wearable actuation [28,44]. By contrast, warp knitting is less represented in rehabilitation robotics but remains attractive because its multi-yarn loop constraint generally produces a more dimensionally stable and mechanically balanced fabric, which is consistent with the common view that warp-knit architectures better support stress redistribution and flatter wearable geometries [44]. The literature here is more mechanistic than comparative, but the logic is consistent: when loop rearrangement is more constrained, local distortion is reduced and the fabric is less likely to develop strongly heterogeneous strain fields during actuation [44].

This same distinction helps explain edge curling suppression. In highly compliant weft-knitted structures, free edges can relax out of plane because the loop network stores and releases deformation asymmetrically when constraints are removed; in actuator design, this can complicate layer alignment, pressure uniformity, and wearable integration [44]. Warp-knit architectures are therefore often considered advantageous not because they necessarily generate larger actuation, but because their stronger inter-yarn constraint can suppress free-edge instability and simplify fabrication of flatter, more stable textile devices [44]. For rehabilitation garments, this can be valuable whenever the priority is sustained body contact, stable pressure distribution, and repeatable actuator alignment rather than maximal strain amplification.

Overall, 3D seamless whole-garment knitting is emerging as one of the most promising textile routes for wearable rehabilitation because it combines seam-free fabrication, anatomical conformity, structural programmability, and integrated sensing-actuation capability in a single platform. At the same time, weft and warp knitting should be treated as distinct mechanical design strategies: weft knitting is better suited to large deformation and compliant actuation, whereas warp knitting appears better suited to stress regularization, flatter geometry, and suppression of edge instability in wearable systems.

### 2.4. Braided, Non-Woven and Other Structures

Beyond woven and knitted structures, braided and non-woven textiles provide additional actuation profiles. Braiding interlaces three or more yarns diagonally to form tubular or rope-like structures. This architecture can translate radial expansion into axial contraction, a principle used in McKibben artificial muscles, which consist of an inner inflatable tube and an outer braided sleeve and were first developed in the 1950s [57]. In textile actuation, braided sleeves are often used as outer shells for pneumatic systems or as reinforcing layers that direct the force of an expanding internal bladder.

Non-woven fabrics are formed by bonding fibers directly, without first converting them into yarns. Although they are less common in actuation fabrics than woven or knitted structures, they are attracting interest in responsive surfaces because they are relatively simple to manufacture and share some features with thin films. In 2024, Zanoni and colleagues showed that non-woven fabrics can amplify photothermally induced reversible actuation compared with bulk films. The fibrous structure enhanced photothermal efficiency through light scattering and thermal insulation, allowing operation at low stress levels close to those of human muscle (0.15–0.40 MPa). The resulting fabric produced greater displacement and showed much better durability under high-frequency cycling than thin films, while remaining lightweight and flexible for wearable applications [58].

In addition to braided and non-woven structures, researchers have explored related architectures such as 3D-printed structures, origami-inspired designs, and fiber-crossing systems [33,59,60,61,62]. Although these approaches differ from traditional textile fabrics, they show how structural design can transform actuation behavior. For example, Zhang and Shea used a fiber-crossing structure to build a mechanics-informed actuator from SMA fibers [33] (Figure 1d). In the single-fiber state, the SMA fibers primarily bend rather than shrink. After assembly into a crossing structure, the bending of individual fibers causes the mesh to contract radially and stretch laterally. This example demonstrates that textile-related structures can not only amplify or limit actuator strain and force but also convert one deformation mode into another to meet application requirements.

Below is a cross-structure comparison (Table 1). Reported values are structure-typical literature ranges, not strictly head-to-head benchmarks, because actuator material, drive mode, and test protocol differ substantially across studies.

### 2.5. Alternative Integration Routes: Sewing, Embroidery, Couching, and Laying-In

Beyond woven, knitted, and braided integration, actuating textiles can also be realized through post-fabrication routes such as sewing, embroidery, couching-like fixation, and laying-in, which introduce actuator fibers or yarns into an already formed textile substrate rather than during fabric construction. This distinction matters because actuation performance depends not only on the active material itself, but also on how the textile architecture constrains, transmits, and spatially organizes deformation across fiber/yarn, fabric, and garment levels [64]. Direct weaving and knitting remain powerful because they make the actuator yarn part of the load-bearing textile network, enabling parallel assembly for force scaling, patterned active/passive regions, reversible 2D-to-3D deformation, and integration of sensing or conductive pathways within the same fabric logic [64,67]. However, these in-process routes also impose geometric and mechanical constraints, since active yarns must tolerate textile manufacture while their actuation is restricted by warp/weft or course/wale placement [69]. For that reason, alternative integration methods are increasingly valuable for actuation-focused textile design. Embroidery, for example, offers much greater spatial freedom than conventional weaving or knitting, because actuator filaments can be stitched along axial, radial, or concentric paths to program localized 2D-to-3D transformations in otherwise passive fabrics [70]. This makes embroidery attractive when actuation must be placed only in selected zones, when complex deformation paths are required, or when an existing functional cloth should retain its original role while gaining motion; thin McKibben artificial muscles, for instance, have been fixed by embroidery onto functional fabrics so that actuator force is transmitted to the cloth while the base textile preserves its native function [71]. More generally, couching-like fixation and laying-in are useful because they secure actuator yarns with less structural commitment than full weaving or knitting, which can reduce disturbance to fragile active elements while still preserving the softness, conformability, and breathability that make textiles attractive for soft robotics and wearables [72]. The tradeoff is that these routes usually provide weaker structural locking than fully integrated textile architectures, but they are often preferable when the design priority is non-destructive implantation, local programmability, or actuator retrofitting onto finished fabrics and garments [70,71].

In summary, the structural topology of actuation textiles—spanning one-dimensional fibers and yarns, two-dimensional woven, knitted, and non-woven fabrics, braided sleeves, as well as alternative post-fabrication integration routes such as embroidery and laying-in—serves as the primary mechanical engine that governs force transmission, deformation anisotropy, strain amplification, and body conformability. While these multi-scale structural configurations dictate how deformation is geometrically routed and mechanically constrained, the ultimate driving force behind macro-scale motion resides in the intrinsic stimulus-responsive behavior of the active building blocks. Therefore, a complete realization of high-performance actuation fabrics requires a synergistic perspective that pairs structural mechanics with material chemistry. The following section systematically examines the key active material classes, categorizing them by their underlying actuation physics, driving mechanisms, and environmental responsiveness.

## 3. Active Materials in Actuation Fabrics

While textile structure determines how an actuation fabric moves and how different components are integrated, the active material provides the driving mechanism. The field has shifted from bulky external motors toward materials that intrinsically change shape in response to stimuli. For use in actuation fabrics, these materials must be sufficiently flexible, tough, and processable to withstand textile manufacturing. They can be broadly classified according to driving mechanism, including thermal, electrical, fluidic, and environmentally responsive systems.

### 3.1. Thermally Driven Materials

Thermally driven materials are among the most widely explored active materials for textile actuation. Representative examples include shape memory alloys (SMAs), especially NiTi alloys, shape memory polymers (SMPs), thermally driven twisted and coiled polymer actuators (TCPAs) based on nylon, PET, PLA, and related polymers, and liquid crystal elastomers (LCEs) as illustrated in Figure 2. When an SMA wire or yarn is integrated into a fabric and heated, often by Joule heating, it returns toward its memorized shape and generates substantial force. For example, Xu et al. fabricated a biomimetic actuator with both shape-memory and color-changing responses using metallic composite yarns and polymeric/thermochromic microcapsule composite fibers. By using a plain-woven structure, they produced a biomimetic dragonfly, demonstrating a design route for multifunctional smart materials [73]. LCEs are another important class of thermally responsive materials. They are soft polymer networks containing liquid crystal mesogens that become disordered upon heating, causing contraction along a programmed axis [74,75]. Compared with metallic actuators, LCEs offer softer and more muscle-like actuation. LCE fibers can be woven or knitted directly into textiles; for example, Wan et al. used LCE fibers to knit tubular fabrics with reversible contraction and morphing behavior [50]. TCPAs also rely on heat-induced contraction or expansion, as demonstrated in the seminal work on fishing-line and sewing-thread artificial muscles [34]. Overall, thermally driven actuation fabrics use heat, including Joule heating, to trigger phase transitions, molecular reorientation, or physical contraction, enabling high output force and adaptive shape transformation.

### 3.2. Electroactive and Ionic Systems

For applications requiring faster response or direct electrical control, researchers have explored electroactive and ionic materials. As shown in Figure 2b, typical systems include electroactive polymers such as dielectric elastomers, ionic polymer-metal composites, conducting polymers, metallic composite yarns, and silver nanowire layers. Conducting polymers, including polypyrrole (PPy) and PEDOT: PSS, actuate through ion migration: when voltage is applied, ions enter or leave the polymer network, causing volume changes. Early systems often required liquid electrolytes, which limited wearable use, but solid-state ionogels have improved air stability and processability. Backe and colleagues developed in-air actuating trilayer tape yarns composed of PEDOT: PSS and ionogel layers, and then integrated them into textile structures by weaving, producing flexible actuation fabrics with large stroke output [19]. Dielectric elastomer actuators (DEAs) provide another route. These systems act as flexible capacitors, in which electrostatic attraction compresses an elastomer and causes area expansion. Zhao’s group developed a wearable haptic communicator based on silicone DEAs, where an elastomer layer was integrated between CNT compliant electrodes to form a Swiss-roll structure mounted on a soft printed circuit board attached to a textile sleeve [76]. Although DEAs can provide rapid, muscle-like actuation, their integration into breathable fabrics remains challenging because they require high-voltage insulation and continuous electrode layers [19].

### 3.3. Fluidic and Pneumatic Systems

**Figure 3:**
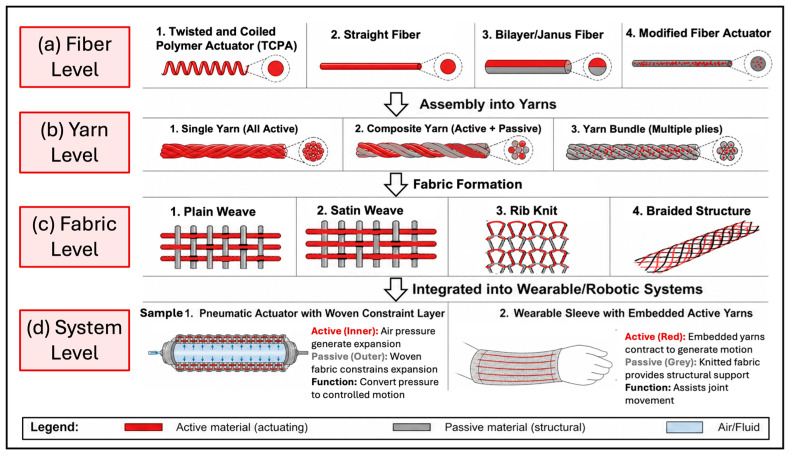
Hierarchical design framework and multi-scale manufacturing routes for smart actuation fabrics, illustrated through a four-level structural integration. (**a**) Fiber Level: Fundamental building blocks and molecular reprogramming. This tier showcases fundamental fiber actuator architectures, including the highly Twisted and Coiled Polymer Actuator (TCPA), Straight Fiber actuators, Bilayer/Janus Fiber architectures, and Modified/Hybrid Fiber Actuators, where fundamental actuation is programmed at the molecular or material composition level. (**b**) Yarn Level: Assembly and structural regulation. Illustrating the assembly of single active fibers into macroscopic yarns. This stage includes (1) Single Twisted Yarns (All Active) for maximum stroke, (2) Composite Yarns combining active and passive materials (e.g., active core/passive sheath or blended twisting), and (3) Yarn Bundles (Multi-Ply) designed for increased output force and mechanical robustness. (**c**) Fabric Level: Architectural programming of deformation. Demonstrating how two-dimensional (2D) textile architectures direct and amplify fiber-level actuation. This level includes stable Plain Weaves, flexible Satin Weaves, highly stretchable Rib Knits for strain amplification, and Braided Structures for multi-axial compliance. (**d**) System Level: Integration into adaptive wearable and robotic interfaces. Showcasing the realization of functional active textiles in real-world applications. Sample 1 illustrates a pneumatic actuator using a woven constraint layer to guide bending (inspired by McKibben-type muscles); Sample 2 demonstrates a wearable sleeve with embedded active yarns providing joint assistance, emphasizing the seamless integration of functional (active) and structural (passive) elements across all multi-scale layers.

Pneumatic and hydraulic systems use a different actuation principle, in which a fluid is confined within a textile shell or bladder. These fabric-based soft pneumatic actuators rely on inflation of an airtight chamber embedded in or laminated onto a fabric. In such systems, the textile is usually passive but structurally essential: anisotropic stiffness guides the direction of expansion as depicted in Figure 2c and Figure 3d. For example, a fabric combining an inextensible woven region with an extensible knitted region can bend toward the woven side when inflated, producing joint-like motion [44]. Fluid-driven fabric actuators can provide high force and high torque-to-weight ratios, making them suitable for rehabilitation exosuits, soft grippers, and other load-bearing soft robotic systems.

As discussed above, fabrics in pneumatic systems often do not serve as active actuation components; instead, they provide structural support that guides the overall deformation. Research on these pneumatic actuators is relatively mature compared with actuators where textile materials are intrinsically active parts. However, emerging filament-shaped McKibben muscles blur this distinction by using textile-like yarns as the pneumatic actuation units [69]. In 2023, Marshall and co-workers created a pneumatic actuation fabric in which the weft yarns themselves were McKibben muscle yarns [63]. This approach illustrates the advantage of actuators based on active textile materials, which can offer more distributed and flexible control than passive constraint fabrics. Their study also characterized the mechanical behavior of plain woven active textiles with different weft muscle densities, providing a basis for more complex fabric designs and applications.

### 3.4. Emerging Responsive Materials

Emerging responsive materials are enabling wireless and more autonomous actuation. Hygroscopic materials, such as natural viscose fibers or carbon nanotube-coated yarns, absorb moisture from the environment and swell (Figure 2d). By designing a fabric in which one side absorbs moisture more strongly than the other, differential swelling can drive curling or bending, similar to the opening of a pinecone. This mechanism is promising for smart clothing that can open ventilation pathways when the wearer sweats. Magnetic actuation provides another tether-free approach (Figure 2e). Reports on using magnetorheological materials as actuators have begun to emerge in top journals [80]. In addition, by embedding magnetic microparticles into yarns or coating fabrics with magnetic composites, textiles can be made to bend, roll, stiffen, or reconfigure rapidly under an external magnetic field [82]. These systems offer possibilities for remote-controlled medical devices and rapidly reconfigurable soft robots.

## 4. System-Level Integration, Grand Challenges, and Future Perspectives

Wearability, washability, and electronics integration are the main barriers to translating actuation fabrics from laboratory prototypes to practical systems. These constraints appear at multiple levels. Nguyen and Zhang showed that fabric soft pneumatic actuators can deliver useful deformation, but their work also makes clear that textile structure, airtightness, and integration strategy remain decisive [29]. Likewise, Sanchez et al. used 3D knitting to build pneumatic soft robotic structures, and du Pasquier et al. developed Haptiknit to combine distributed stiffness knitting with wearable haptics, showing that comfort and function need to be designed together from the outset [24,26]. Even when an active textile delivers strong force or large stroke, it still has to remain breathable, flexible, and comfortable against the skin. At the same time, active fibers and yarns are exposed to abrasion, bending, laundering, and environmental stress, while coatings or encapsulation can improve stability but reduce softness or permeability. Electronics adds another layer of difficulty: Peng et al. noted that power delivery, sensing, and control can quickly dominate device mass when they are treated as add-ons, whereas Kotikian et al. showed that self-sensing liquid-crystal-elastomer actuators can integrate actuation and feedback at the material level rather than relying on later rigid modules [19,60]. Khan et al.’s spider silk supercontraction-inspired cotton–hydrogel self-adapting textiles further suggest that responsiveness and comfort can be embedded directly into soft garment-like substrates [82]. In addition, Wang et al. reported a self-healing MXene/AgNW/liquid metal strain sensor, showing that sensing layers can be built into the textile system with greater robustness than rigid add-on electronics [83].

## 5. Manufacturing Considerations

At the structural level, performance depends on how active fibers are translated into yarns and then into fabrics. Lomov and Huysmans’ hierarchy of textile structures and Wang et al.’s tunable structured fabrics both show that architecture is not passive but part of the actuation mechanism [20,21]. Cappello et al. further demonstrated that textile mechanical anisotropy can be exploited deliberately in fabric-based pneumatic actuators [45]. The same principle applies to actuation fabrics built from SMA, LCE, or conductive yarns: yarn friction, loop geometry, interlacing density, and anisotropy all modify the deformation path, so active materials should be evaluated after textile formation rather than only as isolated strands. Knitted systems usually favor compliance and strain amplification, whereas woven systems tend to provide greater dimensional stability and stronger load transfer [24,29,44,50,53].

More broadly, textile technologies can be selected according to the desired motion and integration route. Sanchez et al. showed that 3D knitting can move pneumatic actuation toward fully textile forms [24], and Atalay et al. demonstrated thermally driven 3D seamless actuation fabrics that better match garment-like geometry [27]. Elmoughni et al. and Ahmed et al. extended this direction to machine-knitted seamless pneumatic actuators and untethered thermally actuated textile exoskeletons, showing that seamless manufacturing can reduce seams, improve fit, and simplify load transfer [54,55]. At the same time, Granberry et al.’s functionally graded knitted actuators and the broader literature on variable-stiffness textile structures suggest that local stiffness, body conformity, and actuation zoning can be programmed directly in the fabric [56,62]. The goal is therefore not to force every actuator into the same architecture, but to match the actuation mechanism to the textile process that best supports it [24,27,54,55,56].

The transition from laboratory demonstrations to practical systems will depend on whether active textiles can be manufactured with existing textile equipment and at reasonable cost. Many current prototypes still rely on hand assembly, limited custom fabrication, or post-processing steps that are difficult to scale. Future work should therefore prioritize process-compatible active yarns, low-resistance textile electrodes, stable coatings, and machine settings that can tolerate fragile functional materials without sacrificing throughput or repeatability. In this context, Zhan et al.’s MXene-functionalized wool yarn artificial muscles show that chemically robust yarns can help bridge the gap between laboratory proof and textile processing [41].

At the same time, scale-up should not be evaluated only at the material level. A high-performance fiber does not automatically become a high-performance fabric after knitting, weaving, or braiding, because structural locking, contact friction, and geometric constraints can change both stroke and force. Active fibers should therefore be benchmarked in their final textile form, where the fabric architecture can reveal whether a given material is truly suitable for wearable actuation. Weaving and braiding also deserve more systematic use as actuation platforms rather than simple carriers. Weaving can provide strong directional constraint and stable load transfer, while braiding is well suited to tubular or muscle-like structures in which fiber angle can be used to tune contraction and expansion. Related architectures such as fiber-crossing, 3D-printed, and hybrid textile systems further show that deformation can be programmed by structural design as much as by material choice.

## 6. Conclusions

Actuation fabrics are emerging as a textile-engineering platform in which motion is generated not only by active materials but also by textile geometry. In this review, we have summarized how woven, knitted, braided, non-woven, and other engineered structures shape the behavior of SMAs, TCPAs, LCEs, electroactive materials, and responsive polymer systems, and how textile architecture can amplify, redirect, or constrain the motion produced by those materials. A central message is that material choice and textile design must be considered together: the same active fiber can exhibit very different force, stroke, and deformation modes after being assembled into different yarn and fabric architectures.

At the same time, the field is still moving toward true multi-scale integration. The examples discussed here show promising routes from fiber- and yarn-level actuation to fabric-level programming through knitting, weaving, braiding, and related construction strategies, but practical deployment still depends on process-compatible active yarns, manufacturing scalability, power supply integration, response speed, durability, and washability. Future progress will therefore require closer coordination between material development, textile processing, and system integration so that actuation fabrics can move from isolated prototypes toward reliable wearable assistance, adaptive garments, soft robotics, and human–machine interaction.

## Figures and Tables

**Table 1:** Concise comparison of representative traditional actuation fabric structures across six common metrics.

Structure	Maximum Contraction Ratio (%)	Output Force/Stress (N or MPa)	Degree of Deformation Freedom	Wear Comfort (Breathability & Compliance)	Fabrication Difficulty	Cyclic Durability (Cycles)
Plain Weave	Low–moderate; typically lower strain than knits, but can be increased by density/pattern design while woven pneumatic systems are strongly design-dependent [18,63]	Moderate–high; weaving is consistently used for force amplification via parallel yarn assembly and strain-limiting layouts [44,64]	Mainly 1D/2D; can be programmed into bending, twisting, and combined 3D motions through multilayer or array design [29,44]	Moderate; high stability and low compliance, but lower elasticity and poorer gas permeability than knits/nonwovens [29,65]	Medium; scalable and mature, but integrating internal channels and complex active architectures remains challenging [51,66]	Moderate–high, but protocol-dependent; woven structures are generally stable and durable, though standardized cycle reporting remains limited [65,66]
Satin Weave	Moderate; expected to exceed plain weave in contractility when longer floats increase yarn freedom, but direct satin-specific values remain sparse [67]	Moderate; force depends strongly on float geometry and weave density, with no robust satin-specific consensus [63,67]	2D to 3D shape morphing; float-rich woven layouts can support more elaborate shape change than plain weave [49,67]	Moderate; more compliant than tightly interlaced weaves, but generally less conformable than knits [67]	Medium–high; performance is sensitive to density and floating balance [63,67]	Insufficient direct evidence; durability is less well documented than for other woven variants [67]
Rib Knit	Moderate–high; knit architectures amplify strain, with a reported 33% contraction in CNT-based knitted actuators [30,53]	Moderate; force transmission is often reduced by loop sliding, though engineered knit-pneumatic systems can still deliver substantial load transfer, including 40 N per actuator [18,26]	Biaxial/multi-DOF/3D; knit mechanics support extension, contraction, bending, and shape change [18,24]	High; knits offer superior compliance, stretchability, elastic recovery, and body conformity [18,29,53]	Low–medium; scalable and programmable, especially with 3D knitting, but property tuning remains nontrivial [24,30]	Moderate; durability is application-dependent and still limited by sliding, stress relaxation, and broader standardization gaps [18,68]
Tubular Braid	Moderate; braided active textiles generally provide greater contraction than a single muscle strand, but usually less strain than knits [18,69]	Moderate–high; braided layouts are favored when contraction force and structural stability are prioritized [69]	Primarily 1D contraction, with broader system-level shape change possible through actuator integration [69]	Moderate; lightweight and flexible, with stability intermediate between knits and dense wovens [18,69]	Medium; fabrication is feasible, but performance depends strongly on braid design parameters [69]	Moderate, but evidence is limited and not standardized across studies [18]
Nonwoven Fabric	Usually low for axial contraction; more commonly used for rapid pneumatic deformation or bending-enabled morphing than large linear shrinkage [44,65]	Low–moderate; limited by poorer strength and elasticity than woven or knitted textiles [44,65]	Mainly 2D/3D morphing, especially in layered pneumatic systems [44,68]	High breathability, but lower structural robustness [65]	Low–medium; easy to process, but requires trade-offs between permeability and mechanical integrity [65]	Low relative to woven/knit structures [65]

## Data Availability

No new data were created or analyzed in this study. Data sharing is not applicable to this article.

## Abbreviations

CNT	Carbon nanotube
DEA	Dielectric elastomer actuator
LCE	Liquid crystal elastomer
PET	Polyethylene terephthalate
PLA	Polylactic acid
SMA	Shape memory alloy
SMP	Shape memory polymer
TCPA	Twisted and coiled polymer actuator
TPU	Thermoplastic urethanes
